# Kisspeptin Neurons and Estrogen–Estrogen Receptor α Signaling: Unraveling the Mystery of Steroid Feedback System Regulating Mammalian Reproduction

**DOI:** 10.3390/ijms22179229

**Published:** 2021-08-26

**Authors:** Yoshihisa Uenoyama, Naoko Inoue, Sho Nakamura, Hiroko Tsukamura

**Affiliations:** 1 Laboratory of Animal Reproduction, Graduate School of Bioagricultural Sciences, Nagoya University, Nagoya 464-8601, Japan; uenoyama@nagoya-u.jp (Y.U.); ninoue@agr.nagoya-u.ac.jp (N.I.); 2 Faculty of Veterinary Medicine, Okayama University of Science, Imabari 794-8555, Japan; s-nakamura@vet.ous.ac.jp

**Keywords:** estradiol, dynorphin A, follicle-stimulating hormone, follicular development, gonadotropin-releasing hormone, luteinizing hormone, *Kiss1*, neurokinin B, ovulation

## Abstract

Estrogen produced by ovarian follicles plays a key role in the central mechanisms controlling reproduction via regulation of gonadotropin-releasing hormone (GnRH) release by its negative and positive feedback actions in female mammals. It has been well accepted that estrogen receptor α (ERα) mediates both estrogen feedback actions, but precise targets had remained as a mystery for decades. Ever since the discovery of kisspeptin neurons as afferent ERα-expressing neurons to govern GnRH neurons, the mechanisms mediating estrogen feedback are gradually being unraveled. The present article overviews the role of kisspeptin neurons in the arcuate nucleus (ARC), which are considered to drive pulsatile GnRH/gonadotropin release and folliculogenesis, in mediating the estrogen negative feedback action, and the role of kisspeptin neurons located in the anteroventral periventricular nucleus-periventricular nucleus (AVPV-PeN), which are thought to drive GnRH/luteinizing hormone (LH) surge and consequent ovulation, in mediating the estrogen positive feedback action. This implication has been confirmed by the studies showing that estrogen-bound ERα down- and up-regulates kisspeptin gene (*Kiss1*) expression in the ARC and AVPV-PeN kisspeptin neurons, respectively. The article also provides the molecular and epigenetic mechanisms regulating *Kiss1* expression in kisspeptin neurons by estrogen. Further, afferent ERα-expressing neurons that may regulate kisspeptin release are discussed.

## 1. Introduction

It has been well accepted that estrogen produced by the ovary plays an indispensable role in the female reproductive system via its feedback actions on gonadotropin-releasing hormone (GnRH) release in mammals. The central mechanisms of the estrogen feedback actions on GnRH release have been a mystery for decades. This is because no report has been available to show the expression of estrogen receptor α (ERα), a critical receptor isoform required for estrogen feedback actions, in the hypothalamic GnRH neurons. Intensive studies on the hypothalamic kisspeptin neurons, which express ERα, have been gradually unraveling the central mechanisms of the negative and positive feedback actions of estrogen on GnRH release. In this article, the physiological significance of the estrogen negative and positive feedback actions on the tonic pulsatile and surge modes of GnRH release—which control folliculogenesis and ovulation in female mammals, respectively—is outlined. Further, the molecular and epigenetic mechanisms mediating the regulation of kisspeptin gene (*Kiss1*) expression by estrogen–ERα signaling and afferent ERα-expressing neurons that may mediate estrogen-dependent modulation of kisspeptin release from the hypothalamic kisspeptin neurons are also discussed.

## 2. Feedback Actions of Estrogen on Pulsatile and Surge-Modes of Gonadotropin-Releasing Hormone (GnRH)/Gonadotropin Release

Mammalian reproduction is orchestrated by the interaction of hormones secreted by the hypothalamus–pituitary–gonadal (HPG) axis. Estrogen secreted from the ovary, downstream of the axis, feeds back to the higher hierarchy hypothalamus and pituitary to regulate GnRH/gonadotropin release. Estrogen production is stimulated by the tonic pulsatile release of gonadotropins, such as luteinizing hormone (LH) and follicle-stimulating hormone (FSH), from the anterior pituitary gland, under the control of GnRH pulses. During the follicular development, circulating estrogen fine-tunes pulsatile release of GnRH to keep circulating levels of LH and FSH adequately. This estrogen action is referred to as “the negative feedback action of estrogen” on GnRH pulses. Under appropriate stimulation by LH and FSH, ovarian follicles develop into a large and mature state. Estrogen production and release gradually increase along with the follicular development, and consequent high levels of circulating estrogen derived from mature follicles (also known as Graafian follicles), in turn, induce a large release of hypothalamic GnRH and then pituitary LH (GnRH/LH surge). This is the so-called “positive feedback action of estrogen” on GnRH release, and the LH surge consequently evokes ovulation. Therefore, the circulating levels of estrogen serve as a messenger for transmitting the maturity status of ovarian follicles to the hypothalamus, which plays a pinnacle role in the hierarchical control of the HPG axis in female mammals.

The presence of hypothalamic GnRH was predicted by Harris and Jacobsohn in the early 1950s by showing that the function of the pituitary graft was restored only when the graft was placed under the median eminence in hypophysectomized rats [1]. This notion was further validated by McCann and colleagues in the early 1960s by showing the LH-releasing ability of hypothalamic extracts in rats [2,3]. The tonic pulsatile LH release, the preovulatory LH surge, and their regulation by estrogen feedback are then clearly demonstrated by Knobil and colleagues from the late 1960s to the early 1970s [4,5,6,7,8]. They demonstrated that tonic LH release was found in most periods of the menstrual cycle, and LH surge was found only in the midcycle before ovulation in humans and rhesus monkeys [4,5]. It was also demonstrated that ovariectomy increased plasma LH concentration, indicating the negative feedback action of some ovarian humoral factor(s) on tonic pulsatile LH release in rhesus monkeys [6]. Importantly, estrogen replacement at a physiological level (not a preovulatory level) suppressed tonic pulsatile LH release in ovariectomized (OVX) rhesus monkeys [8]. These findings suggest that estrogen secreted from the ovary is a major humoral factor that exerts its negative feedback action on the tonic pulsatile LH release. On the other hand, an administration of estrogen at a preovulatory dose induces a vast release of LH similar to the spontaneous preovulatory LH surge, suggesting that the high dose of estrogen exerts its positive feedback action on the LH surge-generating system [7,8]. In 1971, GnRH was isolated from the hypothalamus of pigs and sheep by two groups led by Schally and Guillemin [9,10]. In the early 1990s, Moenter et al. [11,12,13] suggested that GnRH dominantly controls LH release by showing that GnRH pulses and GnRH surge in the pituitary portal circulation were synchronized with LH pulses and LH surge in the peripheral circulation, respectively, in sheep. In addition, Pau et al. [14] showed simultaneous GnRH and LH surges in rhesus monkeys as well. To date, the pulsatile and surge modes of GnRH release have been hypothesized to be driven by the independent hypothalamic mechanisms, so-called “GnRH pulse and surge generators”, respectively [15,16,17]. It is plausible that estrogen regulates the activity of GnRH pulse and surge generators via the negative and positive feedback actions, respectively.

## 3. Indispensable Role of Estrogen Receptor **α** for Mammalian Reproduction

Accumulating evidence indicates that ERα is a critical estrogen receptor isoform responsible for both the negative and positive feedback actions of estrogen on GnRH/LH release. In fact, *Esr1* (coding ERα) knockout mice [18,19] and rats [20] show hypersecretion of both LH and estrogen, indicating that ERα mainly mediates the estrogen negative feedback action. Further, the *Esr1* knockout mice and rats fail to show ovulation, albeit enlarged cystic follicles are found in *Esr1* knockout mice and rats. This suggests that ERα is also responsible for the estrogen positive feedback action [18,19,20]. On the other hand, reproductive function of *Esr2* (coding ERβ, known as another ER) knockout animal models were reportedly varied between animal models: *Esr2* knockout mice showed normosecretion of LH and estrogen [19] and are subfertile with a small litter size [21,22]; *Esr2* knockout rats are infertile with a lack of LH surge and ovulation [23]. In addition, previous studies demonstrated that selective antagonism of estrogen–ERα signaling, but not estrogen–ERβ signaling, eliminated the endogenous LH surge in rats [24]. Taken together, these findings suggest that ERα mainly mediates both estrogen feedback actions on GnRH/LH release.

In general, the ERα is known as a ligand-activated transcriptional factor that activates or represses the expression of target genes. The estrogen-bound ERα is reported to bind to the estrogen response element (ERE) in the target genes to control gene expression [25]. In addition, it is suggested that the estrogen-bound ERα interacts with other transcription factors, such as AP-1 and NF-κB, and the complex controls target gene expression via binding to non-ERE response elements through the transcriptional partner [26,27,28]. Intriguingly, a previous study demonstrated that ERα knock-in/knockout (KIKO) mice, in which a mutant ERα (E207A/G208A) lacks the binding ability for the ERE but is capable of interacting with other transcriptional partners [29,30], showed the negative, but not the positive, feedback action of estrogen on LH release [31]. These findings suggest that the negative feedback action of estrogen on GnRH pulses is likely mediated via some gene(s) controlled by the ERE-independent estrogen–ERα signaling and that the estrogen positive feedback action is likely mediated via some gene(s) controlled by the ERE-dependent estrogen–ERα signaling.

## 4. Possible Targets of the Negative and Positive Feedback Action of Estrogen in the Brain

Precise targets of the negative and positive feedback actions of estrogen on the GnRH pulse and surge generation have been a mystery for many years of the 20th century because no report has been available to show ERα expression in GnRH neurons [32]. Therefore, the most plausible explanations are that certain hypothalamic ERα-expressing cells serve as targets of the negative and positive feedback actions of estrogen on GnRH pulse and surge generation and that such ERα-expressing cells transmit the estrogen signals to GnRH neurons. A large number of ERα-expressing cells were found in the several hypothalamic nuclei—such as the anteroventral periventricular nucleus (AVPV), preoptic area (POA), arcuate nucleus (ARC), and ventromedial nucleus (VMH)—at both the mRNA and protein levels, as well as the paraventricular nucleus (PVN) and suprachiasmatic nucleus (SCN), where ERα expression was evident at the mRNA level in rats [33,34]. Similarly, ERα was mainly found in the POA, ARC, and VMH at both the mRNA and protein levels and in the PVN at the mRNA level in sheep [35,36]. These findings were well consistent with previous studies showing that radiolabeled estrogen was accumulated in the POA, ARC, and VMH in rats [37].

Previous studies suggest that the ARC is one of the most possible targets of negative feedback action of estrogen: Smith and Davidson [38] showed that estrogen implants in the mediobasal hypothalamus (MBH), including the ARC suppressed plasma LH levels in OVX rats in 1974; Akema et al. [39] showed that estrogen implants in the ARC suppressed LH pulses in OVX rats in 1983; furthermore, Nagatani et al. [40] showed that estrogen micro-implants in the ARC suppressed LH pulses in both fasted and re-fed OVX rats, while the estrogen implants in either the PVN or brainstem A2 region suppressed LH pulses in only fasted rats in 1994. These findings suggested that the negative feedback action of estrogen may be mediated by ERα-expressing neurons located in the ARC under the normal nutritional condition and by multiple ERα-expressing neurons located in the ARC, PVN, and brainstem A2 region under the malnutritional condition. The negative feedback actions of estrogen under the malnutritional condition are likely mediated by de novo synthesized ERα in the PVN and brainstem A2 region because 48 h fasting increases the number of ERα-immunoreactive cells in the PVN and brainstem A2 region in OVX rats [41].

Previous studies suggest that ERα-expressing neurons in the AVPV and/or POA are the most possible targets of estrogen positive feedback action: Kawakami et al. [42] and Goodman [43] demonstrated in the late 1970s that estrogen implants into the AVPV or neighboring POA induced the LH surge in OVX rats; Wiegand et al. [44,45] showed in the late 1980s that an electrolytic lesion around the AVPV abolished the estrogen-induced LH surge in OVX rats; Petersen et al. [46,47] demonstrated in the late 1980s that implants of estrogen antagonists, such as LY-10074 or keoxifene, in the AVPV-POA region prevented estrogen-induced LH surge in OVX rats. These reports suggest that the ERα-expressing cells in the AVPV-POA region serve as targets of the estrogen positive feedback actions to induce GnRH/LH surge.

## 5. Kisspeptin Neurons as Targets of the Negative and Positive Feedback Actions of Estrogen

Intensive studies during the past 20 years demonstrate that ERα expression is evident in the hypothalamic kisspeptin neurons in rodents [48,49,50,51] and sheep [52] and that kisspeptin serves as a potent secretagogue of gonadotropin release in rodents [50,53,54,55,56,57,58], ruminants [59,60], and primates [61,62]. To date, it is well accepted that ERα-expressing kisspeptin neurons mainly mediate the estrogen feedback on GnRH release in mammals, and the possible mechanism mediating the feedback effect is discussed in detail later in this article.

Kisspeptin was first discovered as an endogenous ligand for GPR54, an orphan Gq-coupled G-protein coupled receptor (GPCR), in humans in 2001 [63,64]. In 2003, two independent research groups reported that inactivating mutations in the *GPR54* gene caused hypogonadotropic hypogonadism in humans [65,66]. These important findings shed light on the fact that kisspeptin–GPR54 signaling plays a pivotal role in the brain mechanism controlling GnRH/gonadotropin release and then puberty and fertility in mammals [65,66]. As expected, inactivating mutations in the *KISS1* gene (coding kisspeptin) also caused hypogonadotropic hypogonadism in humans [67]. The infertile phenotype in humans carrying inactivating mutations of the *KISS1* or *GPR54* genes was recapitulated in *Kiss1* or *Gpr54* knockout rodent models [66,68,69,70,71,72]. Importantly, *GPR54* expression is evident in GnRH neurons in rodents [54,68,73,74,75], suggesting that kisspeptin directly stimulates GnRH release. Further, *Kiss1* knockout rats show undetectable levels of LH and FSH even after ovariectomy, indicating failure of tonic pulsatile LH release [72]. In addition, the *Kiss1* knockout rats also fail to show estrogen-induced LH surge. These findings suggest that kisspeptin–GPR54 signaling is indispensable for both GnRH pulse and surge generation and mediate feedback actions of estrogen on GnRH/LH release.

**Figure 1:**
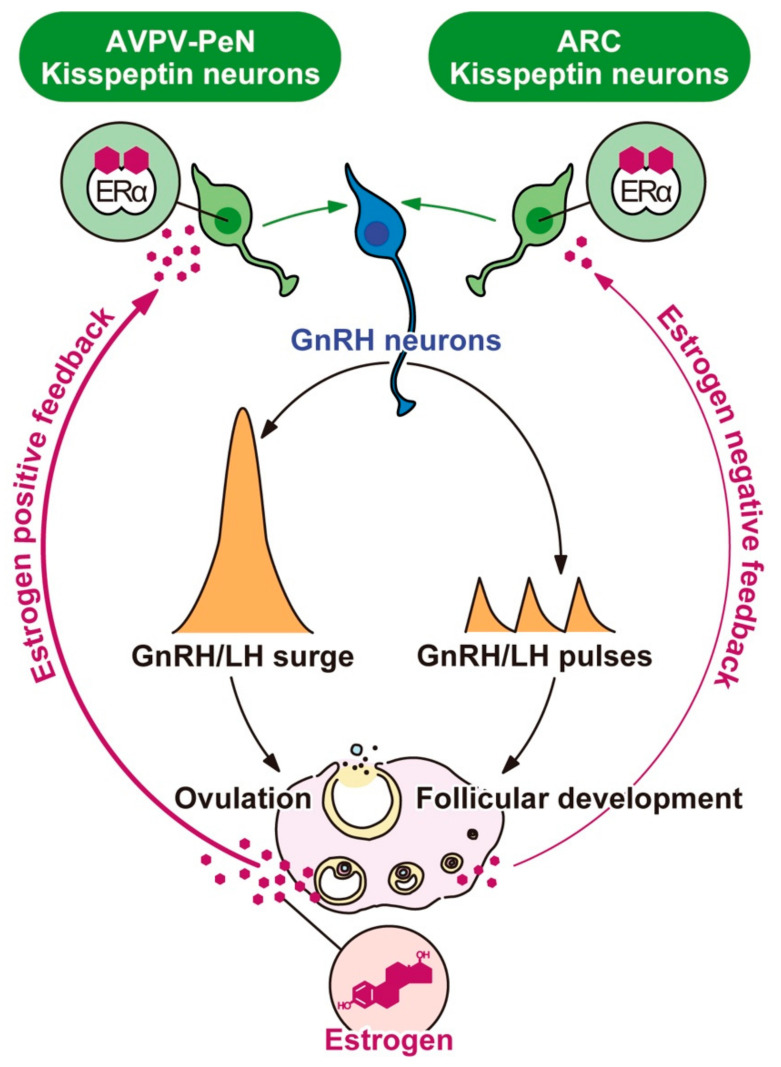
Central mechanisms underlying the negative and positive feedback actions of estrogen on pulsatile and surge modes of gonadotropin-releasing hormone (GnRH)/luteinizing hormone (LH) release in female rodents. Estrogen production along with follicular development is stimulated by GnRH/gonadotropin pulses. During the follicular development period, low levels of circulating estrogen fine-tune GnRH/H pulses via the negative feedback action of estrogen. The estrogen negative feedback action is considered to be mediated by estrogen receptor α (ERα)-expressing kisspeptin neurons located in the arcuate nucleus (ARC). Estrogen production and release gradually increase along with the follicular development, and consequent high levels of circulating estrogen derived from mature follicles, in turn, induce GnRH/LH surge and hence ovulation via the positive feedback action of estrogen. The estrogen positive feedback action is likely mediated by ERα-expressing kisspeptin neurons located in the anteroventral periventricular nucleus–periventricular nucleus continuum (AVPV-PeN).

Histological studies in rodents revealed that cell bodies of kisspeptin neurons are mainly located in the anterior hypothalamic areas, such as the AVPV–periventricular nucleus continuum (AVPV-PeN), and in the posterior hypothalamic region—that is, the ARC [48,49,50,51,76,77,78]. Importantly, ERα was found in both populations of hypothalamic kisspeptin neurons, and *Kiss1* expression is controlled by estrogen in a brain region-specific fashion in rodents [48,49,50,51,76]. More specifically, the ARC *Kiss1* expression level was high at diestrus and was suppressed by estrogen treatment [48,49,50,51,76], whereas the AVPV-PeN *Kiss1* expression level was high at the afternoon of proestrus and was increased by estrogen treatment in rodents [48,49,51,76]. These findings suggest that the ARC kisspeptin neurons are a target of the negative feedback action of estrogen and that the AVPV-PeN kisspeptin neurons are a target of the positive feedback action of estrogen. Figure 1 depicts the brain mechanism mediating the estrogen negative and positive feedback actions on GnRH/gonadotropin release to regulate follicular development and ovulation in rodents. As shown in the figure, it is most likely that the ARC kisspeptin neurons control GnRH/LH pulses via mediating the estrogen negative feedback action and that the AVPV-PeN kisspeptin neurons control GnRH/LH surge via mediating the estrogen positive feedback action.

### 5.1. The Molecular and Epigenetic Mechanism Mediating the Regulation of Arcuate Kiss1 Expression by Estrogen and the Role of arcuate Kisspeptin Neurons as the GnRH Pulse Generator in Mammals

To date, the ARC kisspeptin neurons have been considered to serve as a target of estrogen negative feedback action on GnRH pulse generation, and a similar population of kisspeptin neurons have been identified in the ARC in other species or infundibular nucleus in primates (equivalent to the ARC in others) of several mammalian species, including humans [79,80], macaque monkeys [80,81,82,83], sheep [52,84,85,86], goats [59,87,88], cattle [89], horse [90], pigs [91], and musk shrews [92]. Our studies and other previous studies demonstrated that estrogen treatment largely repressed ARC *Kiss1* expression in rodents [48,49,50,51]. Similar to the rodent models, previous studies demonstrated estrogen-dependent repression of *KISS1* expression in the ARC kisspeptin neurons in sheep [93,94] and the infundibular nucleus in primates including humans [80]. These findings suggest that the estrogen negative feedback action on ARC kisspeptin neurons would be largely common among mammalian species.

According to the studies with rodent models, estrogen-dependent repression of *Kiss1* expression in ARC kisspeptin neurons is likely mediated via the ERE-independent pathway because estrogen repressed the ARC *Kiss1* expression even in ERα KIKO mice [95,96,97]. In addition, our previous chromatin immunoprecipitation (ChIP) assay with antibodies against ERα and acetylated histone H3 revealed that estrogen-bound ERα induced histone H3 deacetylation of the *Kiss1* promoter region in the ARC kisspeptin neurons by showing that estrogen treatment lowered acetylated histone H3 levels in the *Kiss1* promoter region in mouse ARC tissue [98]. These findings suggest that an estrogen-dependent inactivating modification of histone H3 of the *Kiss1* promoter region resulted in the repression of *Kiss1* expression. Furthermore, our in vivo reporter assay utilizing *Kiss1*-GFP reporter mice suggested that the 5′-intergenic region of the *Kiss1* gene is required for an induction of *Kiss1* mRNA expression in the ARC of female mice [99]. Indeed, reporter mice carrying the 5′-truncated *Kiss1*-GFP transgene (RBRC09415 and RBRC09416) failed to display the GFP expression in ARC kisspeptin neurons even after ovariectomy. Importantly, the reporter mice displayed the GFP expression in the AVPV-PeN kisspeptin neurons in the presence of estrogen [99]. Furthermore, other reporter mice carrying the full-length of *Kiss1*-GFP transgene (RBRC09413) displayed the GFP expression in both ARC and AVPV-PeN kisspeptin neurons in OVX and estrogen-treated OVX conditions, respectively [99]. Taken together, we speculate that the estrogen-bound ERα may cancel interaction, which is most likely chromatin loop formation, between the *Kiss1* promoter and 5′-intergenic enhancer regions, resulting in the repression of *Kiss1* expression in ARC kisspeptin neurons even after the ovariectomy.

**Figure 2:**
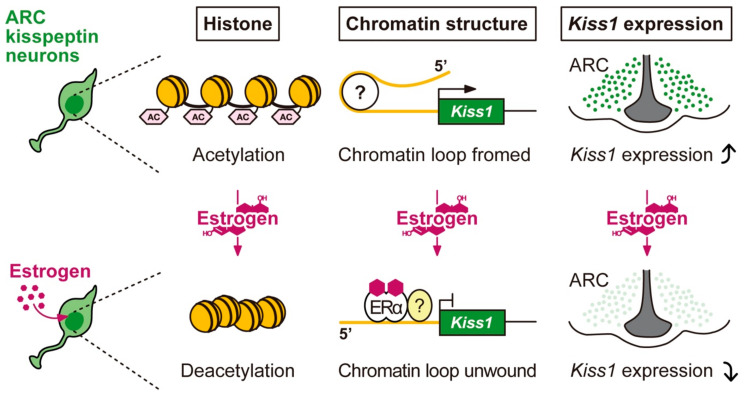
Putative molecular mechanism of the negative feedback action of estrogen on *Kiss1* expression in the arcuate nucleus (ARC). Circulating estrogen seems to act on ARC kisspeptin neurons, in which estrogen-bound estrogen receptor α (ERα) coupled with an unknown transcriptional partner may repress *Kiss1* expression via histone deacetylation and unwinding chromatin loops between the *Kiss1* promoter and the 5′-intergenic regions of *Kiss1* locus. In the absence of estrogen, ARC *Kiss1* expression may be up-regulated by histone acetylation and chromatin loop formation between the *Kiss1* promoter and the 5′-intergenic regions of the *Kiss1* locus.

Collectively, we envisage the molecular mechanism of estrogen negative feedback action on ARC *Kiss1* expression as shown in Figure 2. Briefly, circulating estrogen most likely binds to ERα in the ARC kisspeptin neurons, and then the estrogen-bound ERα coupled with unknown transcriptional partner(s) may repress *Kiss1* expression via a non-classic ERE-independent pathway in ARC kisspeptin neurons. The estrogen-bound ERα may induce histone H3 deacetylation at the *Kiss1* promoter, and the estrogen-bound ERα and/or this inactivating histone modification may unwind chromatin loops between the *Kiss1* promoter and the 5′-intergenic regions of *Kiss1* locus, resulting in the repression of ARC *Kiss1* expression in ARC kisspeptin neurons.

The vast majority of ARC kisspeptin neurons reportedly express neurokinin B (NKB) and dynorphin A (Dyn), thus the ARC kisspeptin neurons are also called KNDy neurons [86,87,100,101,102]. Accumulating evidence suggests that the ARC KNDy neurons can serve as an intrinsic source of the GnRH pulse generator [103,104,105,106,107]. The notion was recently confirmed by our study showing that rescuing *Kiss1* expression only in ARC *Tac3* (NKB gene)-expressing neurons recovered LH pulses and follicular development in global *Kiss1* knockout rats [108]. The multiple-unit activity (MUA) recording demonstrated that rhythmic increases in the MUA volley detected from the recording electrodes placed in close vicinity to ARC kisspeptin (KNDy) neurons were synchronized with LH pulses in goats [59,87]. In addition, conditional ARC-specific *Kiss1* knockout by using the Cre-loxP system severely or partially suppressed LH pulses in rats [108] and mice [109,110] according to the knockout rates in each individual. Further, the fiber photometry recording revealed that the mouse ARC kisspeptin neurons displayed rhythmic increases in intracellular Ca^2+^ levels that correspond to LH pulses [111,112]. Thus, kisspeptin neurons may secrete kisspeptin in a pulsatile fashion and then induce GnRH/gonadotropin pulses. Indeed, Keen et al. [113] and Kurian et al. [114] showed pulsatile kisspeptin release that mostly corresponds to GnRH pulses at the median eminence in rhesus monkeys. Thus, the negative feedback action of estrogen directly acts on the intrinsic source of the GnRH pulse generator—namely, ARC kisspeptin neurons—and then suppresses GnRH/LH pulses. In this context, the profound suppression of GnRH/LH pulses before the afternoon LH surge in the female rodents in the presence of a high dose of estrogen may be due to the abovementioned epigenetic repression of ARC *Kiss1* expression and consequent deficiency of kisspeptin in ARC kisspeptin neurons. Indeed, chronic treatment of preovulatory levels of estrogen profoundly suppresses tonic LH release in the morning (before LH surge) in OVX rats, and the estrogen treatment largely decreased *Kiss1* expression as well as kisspeptin-immunoreactivity in the ARC of female rats [51].

In addition to the direct inhibiting action of estrogen on *Kiss1* expression, estrogen may also inhibit the pulsatile activity of ARC kisspeptin neurons via other intra-kisspeptin neuronal mechanisms or some afferent ERα-expressing neurons to ARC kisspeptin neurons. The frequency of KNDy neuronal activity recorded by the MUA volley was increased and decreased by a central administration of NKB and Dyn, respectively, in goats [87,104]. A majority of KNDy neurons reportedly express both tachykinin NK3 receptor, a Gq-coupled GPCR for NKB, and kappa-opioid receptor (KOR), a Gi-coupled GPCR for Dyn in mice [102,115,116,117], rats [118,119], and sheep [120,121]. Considering the stimulatory or inhibitory signaling of NKB or Dyn, respectively, these findings suggest that the pulsatile activity of ARC kisspeptin (KNDy) neurons is controlled by NKB and Dyn in an autocrine/paracrine manner (please see review articles for details, [103,104,105]). Previous studies showed that estrogen decreased NKB gene (*Tac2* in mice and *Tac3*/*TAC3* in other mammals) expression in the ARC of mice [96,97,122] and sheep [123] and in the infundibular nucleus of rhesus monkeys [124]. In addition, estrogen decreased Dyn gene (*Pdyn*) expression in the ARC of mice [95,96] and rats [125]. These results suggest that estrogen may modulate kisspeptin release from the ARC KNDy neurons via changing stimulatory NKB and inhibitory Dyn inputs to the KNDy neurons.

Interestingly, the proestrous level of estrogen repressed ARC *Kiss1* expression [50,51], whereas the diestrous level of estrogen, which exerted negative feedback action of LH pulses [78], failed to suppress ARC *Kiss1* expression in female rats [50,51]. The dose of estrogen required for the repression of ARC *Kiss1* expression raises the possibility that certain afferent ERα-expressing neurons to ARC kisspeptin neurons may be involved in the negative feedback action of estrogen on kisspeptin release from the ARC kisspeptin neurons. This notion is supported by a previous study showing that estrogen effectively decreased plasma LH concentration even in kisspeptin neuron-specific ERα knockout mice, whose ARC *Kiss1* expression was not repressed by estrogen treatment [126]. One of the candidates mediating the estrogen negative feedback action would be Dyn neurons located in the PVN. Our studies showed that estrogen increased the number of *Pdyn*-expressing cells in the PVN [118] and that nor-binaltorphimine (nor-BNI), a KOR antagonist, enhanced LH pulses in estrogen-treated OVX rats but not in OVX rats without estrogen replacement [127]. Further, our recent study showed that glucoprivation suppressed LH pulses and induced *fos* (coding c-Fos, a marker of neuronal activation) expression in PVN Dyn neurons, while central KOR antagonism blocked the glucoprivic suppression of LH pulses in estrogen-treated OVX rats [118]. These findings suggest that PVN Dyn neurons may partly mediate estrogen negative feedback action to suppress kisspeptin release via KOR expressed in ARC kisspeptin neurons and then suppress pulsatile GnRH/LH release.

### 5.2. The Role of Anteroventral Periventricular Nucleus-Periventricular Nucleus (AVPV-PeN)/Preoptic Area (POA) Kisspeptin Neurons as the GnRH/Luteinizing Hormone (LH) Surge Generator and the Molecular and Epigenetic Mechanism Mediating the Regulation of AVPV-PeN/POA Kiss1 Expression by Estrogen Positive Feedback Action

The AVPV-PeN kisspeptin neurons have been considered to serve as a target of estrogen positive feedback action on GnRH surge generation in rodents, as already mentioned in the article. To date, kisspeptin neurons were found in the POA in several mammalian species, including macaque monkeys [81,83], sheep [85], goats [88], cattle [89], and musk shrews [92], as well as in the PeN in pigs [91]. Previous studies demonstrated that estrogen treatment largely increased AVPV-PeN *Kiss1* expression [48,49,51,128] and induced c-Fos expression in AVPV-PeN kisspeptin neurons in OVX rodent models [49,51]. Similarly, our and other previous studies demonstrated estrogen-induced *KISS1* and/or c-Fos expression in the POA/PeN kisspeptin neurons of macaque monkeys [81,83], sheep [85], goats [88], cattle [89], pigs [91], and musk shrews [92]. Thus, the POA/PeN kisspeptin neurons in those species are likely equivalent to AVPV-PeN kisspeptin neurons in rodents in terms of an estrogen positive feedback action site.

The notion that the AVPV-PeN kisspeptin neurons serve as an intrinsic source of the GnRH surge generator is more verified by the following studies on sex difference in LH surge generation in rodent models [129,130,131,132]. It is well-known that male rats failed to show LH surge even when they were treated with a preovulatory level of estrogen after castration in adulthood [133,134]. Concordantly, male rodents show only a few kisspeptin neurons in the AVPV-PeN even in the presence of estrogen, whereas females exhibit a cluster of AVPV-PeN kisspeptin neurons in the presence of estrogen [51,76,77]. Sex steroids originated from the perinatal testes are considered to cause defeminization of the AVPV-PeN kisspeptin neurons because neonatal castration allowed the estrogen-induced AVPV-PeN *Kiss1* expression and LH surge in genetic male rats in adulthood to be shown [133,134]. In further support, neonatally androgenized/estrogenized female rats displayed the male-like pattern (few) of *Kiss1* expression in the AVPV-PeN and failed to show LH surge in adulthood [76,133]. Thus, these findings suggest that the AVPV-PeN kisspeptin neurons serve as a target of the estrogen positive feedback action and are the intrinsic source of the GnRH surge generator in rodents.

Estrogen-induced *Kiss1* expression in AVPV-PeN kisspeptin neurons is likely mediated via the ERE-dependent pathway because estrogen treatment failed to induce AVPV-PeN *Kiss1* expression and LH surge generation in ERα KIKO mice [31,95]. In addition, our previous study using ChIP assay for ERα and acetylated histone H3 suggested that estrogen-bound ERα binds to the *Kiss1* promoter and enhances histone H3 acetylation of the *Kiss1* promoter region in the AVPV-PeN kisspeptin neurons because estrogen induces ERα binding and histone H3 acetylation at the *Kiss1* promoter region in mouse AVPV-PeN tissue [98]. The finding suggests that an estrogen-dependent activating modification of histone H3 of the *Kiss1* promoter resulted in an induction of *Kiss1* expression. Furthermore, chromatin conformation capture (3C) assay suggested that estrogen induces chromatin loop formation between the *Kiss1* promoter and the 3′-intergenic regions of the *Kiss1* locus in AVPV-PeN kisspeptin neurons in mice [98]. This result also suggests that the 3′-intergenic region of the *Kiss1* locus serves as an enhancer for estrogen-induced *Kiss1* expression in AVPV-PeN kisspeptin neurons. Indeed, our in vivo reporter assay utilizing *Kiss1*-GFP reporter mice suggested that the 3′-intergenic region of the *Kiss1* gene is required for an induction of *Kiss1* mRNA expression by estrogen in the AVPV-PeN of female mice [98]. More specifically, the reporter mice carrying the 3′-truncated *Kiss1*-GFP transgene (RBRC09417) failed to display estrogen-induced GFP expression in AVPV-PeN kisspeptin neurons, but displayed ovariectomy-induced GFP expression in ARC kisspeptin neurons. As described above, the reporter mice carrying the full-length of *Kiss1*-GFP transgene (RBRC09413) display GFP expression in the AVPV-PeN kisspeptin neurons of the OVX mice with estrogen treatment and the ARC kisspeptin neurons without estrogen treatment.

**Figure 3:**
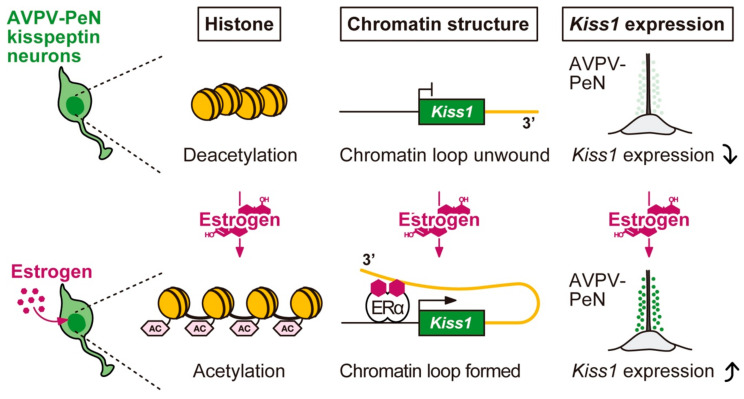
Putative molecular mechanism of the estrogen positive feedback action on *Kiss1* expression in the anteroventral-periventricular nucleus-periventricular nucleus continuum (AVPV-PeN). Preovulatory levels of circulating estrogen seem to act on AVPV-PeN kisspeptin neurons, in which estrogen-bound estrogen receptor α (ERα) may increase *Kiss1* expression via histone acetylation of *Kiss1* promotor region and chromatin loop formation between the *Kiss1* promoter and the 3′-intergenic regions of *Kiss1* locus. In the absence of estrogen, AVPV-PeN *Kiss1* expression may be down-regulated by histone deacetylation of the *Kiss1* promotor region and unwinding chromatin loops between the *Kiss1* promoter and the 3′-intergenic regions of the *Kiss1* locus.

We envisage here the molecular mechanism responsible for the estrogen positive feedback action on AVPV-PeN *Kiss1* expression in rodents, as shown in Figure 3. Briefly, at proestrus in rodents, circulating high levels of estrogen bind to ERα in the AVPV-PeN kisspeptin neurons, and the estrogen-bound ERα may bind to ERE in the *Kiss1* promoter region and enhance histone H3 acetylation at the promoter region. The estrogen-ERα bindings and/or the activating histone modification may form chromatin loops between the *Kiss1* promoter and the 3′-intergenic regions of *Kiss1* locus, resulting in *Kiss1* expression in AVPV-PeN kisspeptin neurons.

Interestingly, both the proestrous and diestrous levels of estrogen are capable of increasing AVPV-PeN *Kiss1* expression in female rats [51], while only the proestrous level of estrogen evoked LH surge in female rats [51]. This fact raises the possibility that, in addition to an increase in *Kiss1* expression in AVPV-PeN kisspeptin neurons, certain afferent ERα-expressing neurons may be also involved in the positive feedback action of estrogen on kisspeptin release from the AVPV-PeN kisspeptin neurons. One of the candidates is brainstem noradrenergic neurons: previous studies showed that ERα expression was found in A2 noradrenergic neurons [135], where estrogen induced c-Fos expression [136], and that α1-adrenergic receptor antagonist attenuated afternoon LH surge in proestrous female rats [137]. Additionally, the SCN, where ERα mRNA expression was found in rats [33], might be also an estrogen positive feedback action site. It is well known that LH surge is timed by the circadian clock localized in the SCN and occurs in the afternoon of proestrus in rodents. A previous study suggested an involvement of SCN vasopressin neurons in the induction of afternoon LH surge because an administration of vasopressin V1 receptor antagonist attenuated afternoon LH surge in proestrous female rats [138]. Interestingly, an electrophysiological study showed that vasopressin treatment induced AVPV-PeN kisspeptin neuronal activity in estrogen-treated OVX mice but not in OVX mice [139], indicating that estrogen may enhance the sensitivity of AVPV-PeN kisspeptin neurons to vasopressin. Taken together, these findings suggest that A2 noradrenergic neurons and SCN vasopressin neurons may mediate estrogen positive feedback to induce kisspeptin release from AVPV-PeN kisspeptin neurons. Previous studies demonstrated that AVPV-PeN kisspeptin neurons largely project their axons to the GnRH cell bodies in the POA in mice [77,140] and that kisspeptin reportedly exerted a long-lasting excitation of GnRH neurons [141,142]. These findings suggest that kisspeptin secreted from the AVPV-PeN kisspeptin neurons may act on GnRH cell bodies to induce GnRH/LH surge.

## 6. Conclusions and Perspectives

Overall, the intensive studies on hypothalamic kisspeptin neurons in the past two decades have been gradually uncovering the cellular and molecular mechanisms of the negative and positive feedback actions of estrogen on GnRH pulse and surge generation in female mammals. Based on the findings currently available, we now postulate that the negative feedback action of estrogen, which fine-tunes GnRH pulses, is mainly mediated by the ARC kisspeptin neurons, in which estrogen directly represses *Kiss1* expression. Further, estrogen may indirectly inhibit pulsatile kisspeptin release via likely afferent ERα-expressing neurons. Further studies are warranted to clarify the afferent inputs that convey estrogen signals to ARC kisspeptin neurons. In addition, we postulate that the positive feedback action of estrogen, which induces GnRH surge, is mainly mediated by the anterior population (the AVPV-PeN in rodents and the POA or PeN in other mammals) of hypothalamic kisspeptin neurons, in which estrogen directly induces *Kiss1* expression. Furthermore, estrogen may indirectly stimulate surge-mode kisspeptin release via likely afferent ERα-expressing neurons, such as brainstem noradrenergic neurons and SCN vasopressin neurons. So far, only a few studies are available to show kisspeptin release, except for the studies in the rhesus monkeys [113,114], as described above. Further studies are needed to depict pulsatile and surge-modes of kisspeptin release and to clarify the mechanism of kisspeptin release controlled by the negative and positive feedback actions of estrogen.

## Data Availability

Not applicable.
